# Late Bowel Symptoms in Long‐Term Survivors of Prostate Cancer Following Radiotherapy

**DOI:** 10.1002/pros.70004

**Published:** 2025-06-24

**Authors:** Isabella Gruber, Oliver Koelbl, Maria M. Meier

**Affiliations:** ^1^ Department of Radiation Oncology University Hospital Regensburg Regensburg Germany; ^2^ University Regensburg Regensburg Germany

**Keywords:** anal canal, bowel symptoms, prostate cancer, prostatectomy, quality of life, radiotherapy

## Abstract

**Background:**

Despite advances in radiotherapy planning, some long‐term prostate cancer survivors experience persistent symptoms. Although overall quality of life appears comparable between patients treated with definitive and salvage radiotherapy and aligns with normative data, prostate‐specific deficits—particularly bowel symptoms—may persist.

**Methods:**

This study assessed prostate‐specific quality of life using the EORTC QLQ‐PR25 questionnaire in 141 patients with localized or locally advanced prostate cancer (T1–4 N0 M0) treated with external radiotherapy between 2011 and 2021. After a median follow‐up of 63.6 months, bowel symptom scores and radiation doses to the anal canal and rectum were analyzed in 71 patients who received definitive radiotherapy (median dose: 78 Gy) and 70 who received salvage radiotherapy (median dose: 70 Gy). Bowel symptom scores were correlated with previously reported global health status scores (EORTC QLQ‐C30) from the same cohort and compared to those of a reference population.

**Results:**

Tumor stage distribution (localized vs. locally advanced) was similar between groups. Patients in the definitive group were older at the time of survey than those in the salvage group (79 years vs. 75 years; *p* = 0.009). Mean doses to the anal canal and rectum were comparable between groups, reflecting consistent application of dose constraints across treatment intents. EORTC QLQ‐PR25 scores, including bowel symptom scores, did not differ significantly between the groups. Patients reporting bowel symptoms (*n* = 92) received significantly higher mean doses to the anal canal (41.0 Gy vs. 35.1 Gy; *p* < 0.001), whereas rectal doses were similar. Mean anal canal dose (*D*mean) correlated with bowel symptom scores (*r* = 0.307; *p* < 0.001), whereas no correlation was observed for mean rectal doses. A *D*mean threshold of 32 Gy to the anal canal differentiated patients with and without bowel symptoms. Higher bowel symptom scores were associated with lower global health status (*r* = −0.469; *p* < 0.001). Compared to the reference population, patients showed significantly and clinically relevant higher bowel symptom scores, indicating a greater symptom burden.

**Conclusions:**

Bowel symptoms are a significant concern after radiotherapy for prostate cancer and are associated with reduced quality of life. Since higher anal canal doses correlate with increased symptom burden, greater efforts to spare this structure during treatment are warranted.

**Trial Registration:**

This study was retrospectively registered.

## 1 Introduction

Radiotherapy is a well‐established treatment for patients with prostate cancer [1]. Advances in radiotherapy techniques, such as intensity‐modulated radiotherapy (IMRT) and volumetric modulated arc therapy (VMAT), have significantly reduced toxicity compared to three‐dimensional radiotherapy by enhancing dose conformity and sparing normal tissues [2]. Despite these advancements, a subset of patients continues to experience persistent symptoms following radiotherapy for prostate cancer [3]. Urinary symptoms are well‐recognized side effects of radiotherapy for prostate cancer, and their association with radiation dose has been examined in previous studies [4]. In contrast, bowel and anal dysfunctions are less well understood and are not routinely reported by patients during follow‐up visits. Therefore, home‐based patient‐reported outcome (PRO) measures may provide a promising method for detecting these symptoms. In a previous study [5], we analyzed health‐related quality of life in long‐term survivors following external radiotherapy for localized and locally advanced prostate cancer, using the European Organization for Research and Treatment of Cancer (EORTC) core questionnaire, EORTC QLQ‐C30 [5]. Although overall quality of life was similar between patients receiving definitive and salvage radiotherapy and matched normative data, bowel symptoms were not adequately reflected in global quality‐of‐life scores.

The present study aims to assess prostate‐specific quality of life using the EORTC QLQ‐PR25 in patients treated with modern radiotherapy techniques, which are known to be associated with fewer side effects compared to conventional three‐dimensional radiotherapy [2]. This study adds to the literature by analyzing the relationship between patient‐reported late bowel symptoms and radiation doses to sensitive organs at risk, such as the anal canal and rectum, following radiotherapy for prostate cancer. Bowel symptoms were also compared to normative data to assess their clinical relevance.

## 2 Materials and Methods

### 2.1 Patients and Data Collection

Between January 2011 and December 2021, a total of 345 patients diagnosed with prostate cancer underwent external radiotherapy at the University Hospital Regensburg. During follow‐up, 42 patients (12.2%) died, whereas 303 patients (87.8%) were still alive. Patients who received radiotherapy for lymph node involvement or distant metastases were not considered for this analysis (*n* = 51). The current place of residence was identified for 247 patients with localized (T1–2 N0 M0) or locally advanced prostate cancer (T3–4 N0 M0), who were then invited to participate in the study via written invitation. EORTC QLQ‐PR25 and QLQ‐C30 questionnaires were sent to 247 patients in April 2023, with final responses received by September 2023. Of these, 145 patients completed the questionnaires, resulting in a response rate of 58.7% (145/247). Four responders who had received adjuvant radiotherapy were excluded from further analysis due to the small sample size of this subgroup. Adjuvant radiotherapy was defined as treatment administered after radical prostatectomy, once the PSA nadir had reached < 0.1 ng/mL. This resulted in a final study population of 141 patients (141/247, 57.1%), comprising 71 patients who received definitive radiotherapy (Group A) and 70 patients who underwent salvage radiotherapy following initial radical prostatectomy (Group B). Salvage radiotherapy was administered to patients who failed to achieve a PSA level below 0.1 ng/mL after radical prostatectomy (persistent PSA) or who experienced a rising PSA level. Clinical data collected included Karnofsky performance status (KPS), patient age at the time of radiotherapy, and age at the time of the survey. Tumor stage and Gleason scores were recorded at the time of prostate cancer diagnosis, along with the PSA level before definitive or salvage radiotherapy. Dosimetric parameters were obtained via chart review, including the volume of the planning target volume (PTV) and the mean (*D*mean) and median doses (*D*50) delivered to the anal canal and rectum.

### 2.2 Cancer Characteristics

Tumor stage at diagnosis (localized vs. locally advanced) was comparable between patients receiving definitive (localized: *n* = 28; locally advanced: *n* = 43) and salvage radiotherapy (localized: *n* = 18; locally advanced: *n* = 52; *p* = 0.106). Similarly, the distribution of Gleason scores did not differ significantly between the groups (*p* = 0.191). Median PSA levels before treatment were 7.9 ng/mL (interquartile range [IQR]: 4.8–13.2) for definitive radiotherapy and 0.3 ng/mL (IQR: 0.2–0.7) for salvage radiotherapy.

### 2.3 Radiotherapy

From 2011 to 2013, two Siemens Primus linear accelerators (Siemens Medical Systems Inc., Concord, CA) were used, and from 2013 to 2021, two linear accelerators of type Elekta Synergy with an Agility head (Elekta Ltd, Crawley, UK). All patients were instructed to maintain a consistently full bladder and an empty rectum before computed tomography (CT) planning and each daily radiotherapy session. To ensure precise and reproducible patient positioning, immobilization was achieved using a blue bag vacuum cushion, which created a customized mold conforming to each patient's body contour. Magnetic resonance imaging (MRI) was fused with planning CT scans to delineate the prostate and seminal vesicles in the definitive setting and to localize the initial anatomical positions of these structures in the salvage setting following prostatectomy. In the definitive radiotherapy setting, the clinical target volume (CTV) encompassed the entire prostate gland and seminal vesicles [6]. In the salvage radiotherapy setting following radical prostatectomy, the CTV included the prostate bed, as defined by consensus guidelines [7], and was extended to encompass regions considered at increased risk of microscopic disease based on pathological findings. The PTV was generated by expanding the CTV using an institution‐specific margin, ranging from 5 to 8 mm, to account for setup uncertainties and intra‐fractional motion. A reduced margin of 5 mm was applied posteriorly toward the rectum to minimize dose to adjacent organs at risk. The median doses administered were 78.0 Gy (IQR: 77.9–78.0 Gy) for definitive radiotherapy and 70.0 Gy (IQR: 70.0–70.0 Gy) for salvage radiotherapy.

Organs at risk included the bladder, rectum, anal canal, small intestine, bulbus of the penis, and femoral heads. Dose constraints have been defined for the anal canal (mean dose [*D*mean] < 40 Gy), bladder (dose to 50% volume [*D*50%] < 65 Gy), rectum (dose to 50% volume [*D*50%] < 50 Gy), femoral heads (maximum dose to 0.2 cc [*D*0.2cc] < 50 Gy), the small intestine (maximum dose to 0.2 cc [*D*0.2cc] < 55 Gy and dose to < 100 cc < 40 Gy), and volume receiving 90 Gy (V90 Gy) < 50% for the bulbus of the penis. MRI scans were fused with CT images to enhance the accuracy of contouring for the prostatic apex and anal canal. The anal canal was delineated below the lowest section of a visible rectal lumen, typically corresponding to the levator ani muscle, to the anal verge. Treatment during the study period was delivered 5 days per week using linear accelerators with IMRT and VMAT techniques, employing 6 megavoltage (MV) photon beams. Patient positioning and the filling of organs at risk during radiotherapy were routinely verified using kilovoltage (kV) cone beam computed tomography (CBCT) as part of image‐guided radiotherapy (IGRT) techniques. This approach ensured accurate and consistent treatment delivery throughout the course of therapy.

A total of 24 patients received androgen deprivation therapy (ADT) during radiotherapy—11 in the salvage radiotherapy group and 13 in the definitive group. Patients attended their first follow‐up visit 6 weeks after completing treatment, followed by visits every 6 months for 2 years, and annually thereafter. Urological follow‐up, including PSA monitoring, was conducted concurrently by a urologist.

### 2.4 Questionnaires

The prostate cancer‐specific EORTC QLQ‐PR25 is designed for patients with prostate cancer and consists of multi‐item scales analyzing bowel symptoms, urinary symptoms, sexual activity, sexual functioning, and hormonal‐treatment‐related symptoms, along with a single‐item scale analyzing incontinence aid use [8]. Higher scores on the functional scales (sexual activity and sexual functioning) indicate better functioning, whereas higher scores on the bowel, urinary, hormonal‐treatment‐related symptoms, and incontinence items reflect more severe problems [8]. All patients rated the extent to which they experienced symptoms during the past week using a 4‐point Likert scale (1 = *not at all*, 2 = *a little*, 3 = *quite a bit*, and 4 = *very much*). For scoring, the average of the items (raw score) was calculated, and these raw scores were linearly transformed into a 0–100 range for both functional and symptom scales [8]. The bowel symptom scale included the following items: (1) limitation of daily activities due to bowel problems, (2) unintentional stool leakage, (3) presence of blood in stools, and (4) abdominal bloating. The bowel symptom scales were dichotomized into two categories: “*not at all*,” corresponding to a bowel symptom scale score of 0, and “*to some extent*,” corresponding to a score greater than 0. Four bowel symptom scores were set to missing. Bowel symptom scales were correlated with previously published data [5], specifically the global health status scales from the EORTC QLQ‐C30 [9]. A higher score on the global health status scale indicated a higher quality of life [9].

The study compared the bowel symptom scales of the study population with reference values from the US population [10], as no European reference values were available at the time of analysis. Cella et al. provided normative data for the US population across six PRO questionnaires, including the EORTC QLQ‐PR25 [10]. The male reference sample (*N* = 876) was age‐adjusted to reflect the demographic characteristics of participants in prostate cancer trials, with a mean and median age of 67.5 and 70.0 years, respectively. The majority of the sample were White and non‐Hispanic [10]. The mean (standard deviation [SD]) reference value for bowel symptoms was 4.7 (11.4) [10].

The local Ethics Board of the University Regensburg approved this study (approval number, 23‐3287‐101, date March 29, 2023). Written informed consent was obtained from all participants.

### 2.5 Statistical Analysis

Patient characteristics are presented as medians with IQRs, means with SDs for continuous variables, and as absolute and relative frequencies for categorical variables. Categorical variables were analyzed using the chi‐square test of independence. Continuous variables were compared using the Mann–Whitney *U*‐test. The Spearman rank correlation coefficient (*r*) was calculated to assess the relationship between variables. Statistical differences and clinical relevance were evaluated between patient data and normative values. A difference of 0.5 SDs was used to determine the clinical relevance of PRO measures. A two‐sided *p*‐value of < 0.05 was considered statistically significant. Statistical analyses were conducted using SPSS version 29.0 (SPSS Inc., Chicago, IL, USA).

## 3 Results

### 3.1 Patient Characteristics

Seventy‐one patients underwent definitive radiotherapy (Group A), and 70 patients received salvage radiotherapy (Group B) for prostate cancer. The median follow‐up period from the last day of radiotherapy to the return of questionnaires was 63.6 months (IQR: 34.7–92.7 months). The median follow‐up duration did not differ significantly between the definitive radiotherapy group (65.2 months, IQR: 37.4–89.1 months) and the salvage radiotherapy group (60.3 months, IQR: 33.2–95.1 months; *p* = 0.743). Table 1 presents the patient characteristics and dosimetric data. Patients treated with definitive radiotherapy were older than those treated with salvage radiotherapy, both at the time of treatment (72 years vs. 69 years; *p* = 0.007) and at the time of the survey (79 years vs. 75 years; *p* = 0.009). The mean doses to the anal canal (*p* = 0.783) and rectum (*p* = 0.544) were similar between the definitive and salvage radiotherapy groups, as organ‐at‐risk dose constraints are applied consistently regardless of treatment intent or total dose (Table 1).

**Table 1:** Patient characteristics and dosimetric data.

	All (*n* = 141)	Definitive radiotherapy group A (*n* = 71)	Salvage radiotherapy group B (*n* = 70)	*p*‐value definitive vs. salvage
Patient age at RT				0.007**
Median (IQR)	70 (64–75)	72 (66–77)	69 (63–72)	
Patient age at survey^a^				0.009**
Median (IQR	75 (70–82)	79 (72–83)	75 (70–80)	
KPS at RT				0.921
Median (IQR)	90 (80–90)	90 (80–90)	90 (80–90)	
PTV^b^ (cc)				0.136
Median (IQR)	307.0 (247.8–376.8)	292.0 (246.8–361.7)	330.5 (249.4–391.1)	
Anal canal volume (cc)				0.283
Median (IQR)	13.9 (8.8–18.0)	13.8 (9.5–18.0)	14.2 (8.0–18.0)	
Rectal volume (cc)				0.820
Median (IQR)	64.2 (45.8–79.8)	62.0 (41.5–80.9)	67.1 (50.7–79.0)	
RT doses (Gy) to the anal canal				0.783
*D*mean (IQR)	38.1 (31.3–46.8)	38.1 (31.0–47.1)	38.5 (32.2–46.0)	
RT doses (Gy) to the rectum				0.544
*D*mean (IQR)	40.8 (34.8–46.9)	40.8 (34.1–46.5)	41.0 (35.2–47.1)	

*Note:* Definitive radiotherapy was administered at a median dose of 78.0 Gy (IQR: 77.9–78.0 Gy). Salvage radiotherapy was delivered at a median dose of 70.0 Gy (IQR: 70.0–70.0 Gy).

Abbreviations: Gy, Gray; IQR, interquartile range; KPS, Karnofsky performance status; RT, radiotherapy.

^a^ The EORTC QLQ‐PR25 questionnaires were completed by patients after a median follow‐up period of 63.6 months (IQR: 34.7–92.7 months) following radiotherapy.

^b^ The planning target volume (PTV) was created by expanding the clinical target volume (CTV) by 5–8 mm. In the definitive group, the CTV included the prostate and seminal vesicles; in the salvage group, it covered the prostate bed and areas at risk of microscopic disease based on pathology.

*p < 0.05, **p < 0.01, and ***p < 0.001.

The studied patient population (*n* = 141) and the excluded population—which comprised nonresponders as well as those excluded due to adjuvant treatment intent (*n* = 106)—were comparable in terms of age at the time of radiotherapy (*p* = 0.632). Among excluded patients (*n* = 106), the median age at radiotherapy was 69 years (IQR: 64–75). The distribution of treatment intent (salvage vs. definitive radiotherapy) was also similar between the studied population and the excluded population (salvage: *n* = 70 vs. *n* = 43; definitive: 71 vs. *n* = 50; *p* = 0.689). The median KPS among excluded patients was 90 (IQR: 80–90), which did not differ significantly from that of the included patients (*p* = 0.543). The median PTV in excluded patients was 288.3 cc (IQR: 226.9–351.7), with no significant difference compared to the included population (*p* = 0.091). Similarly, mean (*p* = 0.507) and median (*p* = 0.200) radiation doses to the anal canal, as well as mean (*p* = 0.339) and median (*p* = 0.957) doses to the rectum, did not differ significantly between the groups.

### 3.2 EORTC QLQ‐PR25 Scales

The EORTC QLQ‐PR25 scales, which assess urinary, bowel, and hormonal‐treatment‐related symptoms, as well as sexual activity and sexual functioning, were similar between patients in the definitive and salvage radiotherapy groups (Table 2).

**Table 2:** EORTC QLQ‐PR25 symptom and functional scales in patients following definitive and salvage radiotherapy for prostate cancer.

EORTC QLQ‐PR25 range 0–100	All	Definitive radiotherapy Group A	Salvage radiotherapy Group B	*p*‐value definitive vs. salvage
*Symptom scales* a				
Bowel symptoms (*n* = 137)				0.108
Mean (SD) Mean Median (IQR)	14.7 (17.2) 8.3 (0.0–25.0)	17.6 (19.8) 8.3 (0.0–25.0)	11.7 (13.7) 8.3 (0.0–16.7)	
Urinary symptoms (*n* = 139)				0.173
Mean (SD) Median (IQR)	28.7 (20.4) 25.0 (12.5–41.7)	27.3 (21.8) 25.0 (8.3–37.5)	30.2 (18.8) 25.0 (16.7–45.8)	
Incontinence aid (*n* = 50)				0.882
Mean (SD) Median (IQR)	38.0 (33.7) 33.3 (0.0–66.7)	40.7 (36.4) 33.3 (0.0–66.7)	37.4 (33.5) 33.3 (0.0–66.7)	
Hormonal‐treatment‐related symptoms (*n* = 137)				0.371
Mean (SD) Median (IQR)	17.1 (13.6) 16.7 (5.6–22.2)	15.8 (12.5) 16.7 (5.6–22.2)	18.4 (14.6) 16.7 (5.6–30.6)	
*Functional scales* b				
Sexual activity (*n* = 136)				0.259
Mean (SD) Median (IQR)	74.7 (24.4) 83.3 (66.7–100.0)	73.3 (23.1) 66.7 (66.7–100.0)	76.2 (25.8) 83.3 (66.7–100.0)	
Sexual functioning (*n* = 41)				0.769
Mean (SD) Median (IQR)	48.9 (18.9) 50.0 (33.3–55.5)	48.3 (15.7) 50.0 (33.3–50.0)	49.6 (22.5) 50.0 (41.7–58.3)	

*Note:* The EORTC QLQ‐PR25 questionnaires were completed by patients after a median follow‐up period of 63.6 months (IQR: 34.7–92.7 months) following radiotherapy. Definitive radiotherapy was administered at a median dose of 78.0 Gy (IQR: 77.9–78.0 Gy). Salvage radiotherapy was delivered at a median dose of 70.0 Gy (IQR: 70.0–70.0 Gy).

Abbreviations: IQR, interquartile range; SD, standard deviation.

^a^ A high score for the symptom scales represents a high level of problems.

^b^ A high score for the functional scales represents a high level of functioning.

*p < 0.05, **p < 0.01, and ***p < 0.001.

### 3.3 Relationship Between Organ‐at‐Risk Doses and Bowel Symptom Scores

Since the mean and median doses delivered to the anal canal and rectum were similar between the definitive and salvage radiotherapy groups—due to identical dose constraints—the relationship between organ‐at‐risk doses and bowel symptom scales was analyzed for both groups combined. A total of 65.2% (*n* = 92) of patients reported bowel symptoms to some extent. Higher mean doses to the anal canal were significantly associated with increased bowel symptom scores (*r* = 0.307; *p* < 0.001***), whereas no association was observed for mean rectal doses (*r* = 0.013; *p* = 0.879).

Table 3 compares the mean doses (*D*mean) delivered to the rectum and anal canal in patients with bowel symptoms (bowel symptom score > 0) and those without bowel symptoms (bowel symptom score = 0). Rectal doses (*D*mean) were similar between patients with and without bowel symptoms. In contrast, patients with bowel symptoms (bowel symptom score > 0) received significantly higher mean anal canal doses compared to those without bowel symptoms (mean: 41.0 Gy vs. 35.1 Gy; *p* < 0.001***) (Table 3). The observed differences in symptoms were not attributable to variations in follow‐up, as the median follow‐up was similar between patients with bowel symptoms (63.1 months, IQR: 32.6–88.8 months) and those without symptoms (64.4 months, IQR: 38.9–100.9 months; *p* = 0.319).

**Table 3:** Radiotherapy doses to the rectum and anal canal in patients with and without bowel symptoms.

Radiation doses (Gy)	EORTC QLQ‐PR25 Bowel symptoms of some extent^a^ (*n* = 92)	EORTC QLQ‐PR25 Bowel symptoms not at all^b^ (*n* = 45)	*p*‐value
Mean dose to the rectum			0.190
*D*mean (IQR)	42.5 Gy (36.9–47.0 Gy)	39.4 Gy (34.7–45.2 Gy)	
Mean dose to the anal canal			< 0.001***
*D*mean (IQR)	41.0 Gy (34.4–47.8 Gy)	35.1 Gy (28.3–39.4 Gy)	

*Note:* The EORTC QLQ‐PR25 questionnaires were completed by patients after a median follow‐up period of 63.6 months (IQR: 34.7–92.7 months) following radiotherapy. Four EORTC QLQ‐PR25 bowel symptom scores were set to missing.

Abbreviations: Gy, Gray; IQR, interquartile range.

^a^ “Bowel symptoms of some extent” correspond to an EORTC QLQ‐PR25 bowel symptom score > 0.

^b^ “Bowel symptoms not at all” correspond to an EORTC QLQ‐PR25 bowel symptom score of 0.

*p < 0.05, **p < 0.01, and ***p < 0.001.

A mean dose (*D*mean) threshold of 32 Gy to the anal canal (*p* = 0.014) appeared to differentiate between patients with (*n* = 74, 73.3%) and without (*n* = 27, 26.7%) bowel symptoms.

### 3.4 Relationship Between the EORTC QLQ‐C30 Global Health Status Score and Bowel Symptom Scores

As previously published in *Scientific Reports* [5], global health status scores (EORTC QLQ‐C30) were comparable between the salvage (mean: 67.5, SD: 23.0, median: 75.0, IQR: 50.0–83.3) and definitive radiotherapy groups (mean: 72.4, SD: 20.2, median: 75.0, IQR: 66.7–83.3; *p* = 0.264).

Global health status was significantly lower (*p* < 0.001***) in patients with bowel symptoms (mean: 64.9, SD: 21.9, median: 66.7, IQR: 50.0–83.3) compared to those without bowel symptoms (mean: 78.7, SD: 18.5, median: 83.3, IQR: 66.7–91.7). Therefore, higher bowel symptom scores were strongly associated with lower global health status (*r* = −0.469, *p* < 0.001***).

### 3.5 Comparison of Bowel Symptom Scores With Reference Values

The patient population had statistically significant (*p* < 0.001***) higher bowel symptom scores (mean: 14.7, SD: 17.2) than the reference population (mean: 4.7, SD: 11.4) [10], indicating greater bowel problems in the patient group. This difference was clinically relevant.

## 4 Discussion

In a previous study [5], we analyzed health‐related quality of life in the same patient group following definitive and salvage radiotherapy for prostate cancer using the EORTC QLQ‐C30. This analysis revealed no significant difference in overall health‐related quality of life between patients who received definitive radiotherapy and those who underwent salvage radiotherapy after radical prostatectomy [5]. However, prostate‐specific symptoms, such as bowel and anal symptoms, are not captured by the EORTC QLQ‐C30. To further explore patient‐reported bowel and anal symptoms, this study assessed prostate‐specific quality of life using the EORTC QLQ‐PR25, in addition to the EORTC QLQ‐C30, in these long‐term cancer survivors at the same time point.

Literature suggests that the anal canal plays a distinct role in the development of late radiation‐induced toxicity [11]. However, late anal and bowel dysfunction is often overlooked during routine follow‐up visits or remains unreported by patients. Therefore, home‐based PRO measures appear to be a promising method for detecting these symptoms.

With a median follow‐up period of 64 months (approximately 5 years)—defined as the interval between completion of radiotherapy and the assessment of PRO measures—the reported symptoms are considered to reflect the late effects of radiotherapy. The study found that prostate‐specific quality of life, including bowel symptoms, was comparable between the treatment groups. Of note, mean doses to the anal canal and rectum were comparable in both the definitive and salvage radiotherapy groups, as organ‐at‐risk dose constraints were consistently applied regardless of treatment intent or total radiation dose. However, 65% of patients reported bowel symptoms to some extent, and radiation doses to the anal canal—but not to the rectum—were associated with late bowel symptoms. A mean dose threshold of 32 Gy to the anal canal appeared to distinguish between patients with and without bowel symptoms. These findings suggest that radiation dose to the anal canal, rather than to the rectum, is a key factor in the development of late bowel symptoms.

This study further explored whether bowel symptoms were associated with a lower global health status. Patients who experienced bowel symptoms reported significantly lower global health status scores compared to those without bowel symptoms. Additionally, when comparing the bowel symptoms of the patient population to normative data of Cella et al. a clinically significant higher frequency of bowel symptoms was observed in the patient group compared to the reference population [10]. As the reference population in the study by Cella et al. [10] appears comparable in terms of patient age to the present cohort, the higher bowel symptom burden observed in our study is likely to be genuine. These findings underscore the detrimental impact of bowel symptoms on overall quality of life. Therefore, efforts should be made to minimize radiation exposure to the anal canal, even when established dose constraints are met.

These findings align partly with those of other studies investigating long‐term outcomes following radiotherapy for prostate cancer [3, 12]. However, some studies have reported radiation doses to organs at risk and clinical outcomes based on older radiotherapy techniques, such as conventional three‐dimensional conformal radiotherapy, which typically exposes surrounding normal tissues to higher radiation doses. In contrast, the present study contributes to the literature by analyzing the radiation doses to the anal canal and rectum following the use of modern radiotherapy techniques, which are associated with fewer side effects due to improved sparing of the bladder, rectum, and anal canal [2]. Mols et al. [12], using conventional three‐dimensional radiotherapy, analyzed bowel and urinary symptoms in 780 prostate cancer survivors 5–10 years posttreatment using the Expanded Prostate Cancer Index (EPIC) questionnaire. Urinary function was significantly worse after prostatectomy, whereas bowel function, bother, and summary scores were significantly worse after radiotherapy [12]. The Prostate Testing for Cancer and Treatment (Protect T) study examined PROs 12 years after treatment, comparing prostatectomy alone, active monitoring, and three‐dimensional conformal radiotherapy [3]. Urinary leakage persisted in the prostatectomy group, whereas fecal leakage (≥ once/week) was twice as common after radiotherapy (12%) compared to the other groups (6%). Despite this, the impact of bowel symptoms on quality of life was similar across groups between Years 7 and 12. Bloody stools, initially more frequent after radiotherapy, resolved over time and aligned with prostatectomy rates during the 7–12 year follow‐up period [3].

Some studies have reported an association between radiotherapy for prostate cancer and anal dysfunction posttreatment, with some of these studies utilizing radiotherapy techniques primarily based on three‐dimensional approaches. Smeenk et al. [13] observed reduced anal pressures following radiotherapy for prostate cancer, identifying a mean dose (*D*mean) > 38 Gy to the anal wall as a predictor of urgency. This study used three‐dimensional conformal radiotherapy, or five‐field IMRT [13]. Buettner et al. [11] conducted a multicenter randomized controlled trial involving 388 patients with a median follow‐up of 24 months. They found a significant positive correlation between sphincter‐related symptoms and the dose received by the anal canal wall. The authors recommended that mean doses to the anal sphincter be limited to 30 Gy or less to reduced loss of sphincter control. Similarly, Peeters et al. [14, 15] confirmed that the mean dose to the anal wall is a strong predictor of fecal incontinence following treatment with various three‐dimensional conformal radiotherapy techniques.

This study focused on mean radiation doses to the anal canal volume, rather than the maximum dose, as the latter represents a point dose within the organ. This is in line with other reports [16]. Onjukka et al. [16] analyzed anorectal dose maps and the risk of late toxicity after IMRT for prostate cancer, reporting that the irradiated volume of the anal canal, rather than the maximum dose, is a significant risk factor for fecal incontinence [16]. These findings underscore the importance of minimizing the radiation dose to the anal canal volume to reduce the risk of stool leakage and other related complications.

At our institution, the dose constraint for the anal canal was a mean dose (*D*mean) of less than 40 Gy. Given that 65% of patients reported bowel symptoms, these findings suggest that additional dose‐reduction strategies for the anal canal should be considered. Reducing the radiation dose to the anal canal can be achieved by limiting the inferior extent of the PTV, while still ensuring adequate coverage of the prostatic apex. The routine use of MRI is strongly recommended for accurately delineating the inferior border of both the prostatic apex and anal canal [17]. In summary, PROs should be integrated into toxicity assessment [18], as not all symptoms are captured during direct clinical encounters.

The primary aim of this study was to analyze patient‐reported prostate‐specific quality of life following radiotherapy for prostate cancer. As such, the study lacked a control group of patients treated with active monitoring or prostatectomy alone. Additionally, the study did not assess dose distributions to subregions of the anal canal and rectum, which may limit the generalizability of the findings. Baseline symptoms and pretreatment organ status were not recorded, which may have introduced bias or confounded the results. Furthermore, the study was based on planned radiotherapy doses rather than the actual delivered doses. However, given the anal canal's fixed position with minimal motion and a consistent circumference, this approach was deemed reasonable. The impact of ADT and PSA relapses following radiotherapy was outside the scope of this study and, therefore, was not analyzed. Similarly, the effect of bladder doses on urinary symptoms was not addressed, as this has been explored in other studies. Lastly, since only 57.1% of all treated patients responded, it is uncertain whether the results can be generalized to the entire unselected patient population. Moreover, based on the available data, we were unable to identify any reasons for the substantial nonresponse rate.

## 5 Conclusions

A significant proportion of long‐term cancer survivors experienced bowel symptoms following modern radiotherapy for prostate cancer, regardless of treatment intent. Patients reporting bowel symptoms received higher doses to the anal canal compared to those without bowel symptoms. A significant negative correlation was observed between bowel symptoms and global health status, with patients reporting any bowel symptoms exhibiting substantially lower global health status scores. These findings emphasize the detrimental impact of bowel‐related side effects on overall quality of life. Consequently, the anal canal should be carefully delineated as a separate organ at risk and spared in pelvic radiotherapy to minimize the risk of late toxicity. Bowel symptoms are not only a concern in radiotherapy for prostate cancer but also in treatments for rectal, vulvar, vaginal, and endometrial carcinomas, where similarly high doses may be delivered to the anal canal. The findings of this study advocate for the adoption of additional dose‐reduction strategies for the anal canal in pelvic radiotherapy, potentially improving patient outcomes and quality of life.

## Disclosure

Permission to reproduce material from other sources. No material from other sources was used in this manuscript.

## Ethics Statement

The local Ethics Board of the University Regensburg approved this study (approval number, 23‐3287‐101, date March 29, 2023). The study was conducted according to the guidelines of the Declaration of Helsinki.

## Consent

All patients gave written informed consent.

## Conflicts of Interest

The authors declare no conflicts of interest.

## Data Availability

The data that support the findings of this study are available on request from the corresponding author. The data are not publicly available due to privacy or ethical restrictions.
